# Automatic segmentation of white matter hyperintensities in routine clinical brain MRI by 2D VB-Net: A large-scale study

**DOI:** 10.3389/fnagi.2022.915009

**Published:** 2022-07-29

**Authors:** Wenhao Zhu, Hao Huang, Yaqi Zhou, Feng Shi, Hong Shen, Ran Chen, Rui Hua, Wei Wang, Shabei Xu, Xiang Luo

**Affiliations:** ^1^Department of Neurology, Tongji Hospital, Tongji Medical College, Huazhong University of Science and Technology, Wuhan, China; ^2^Shanghai United Imaging Intelligence, Wuhan, China; ^3^Shanghai United Imaging Intelligence, Shanghai, China

**Keywords:** white matter hyperintensities, segmentation, brain MRI, convolutional neural network, multi-modality

## Abstract

White matter hyperintensities (WMH) are imaging manifestations frequently observed in various neurological disorders, yet the clinical application of WMH quantification is limited. In this study, we designed a series of dedicated WMH labeling protocols and proposed a convolutional neural network named 2D VB-Net for the segmentation of WMH and other coexisting intracranial lesions based on a large dataset of 1,045 subjects across various demographics and multiple scanners using 2D thick-slice protocols that are more commonly applied in clinical practice. Using our labeling pipeline, the Dice consistency of the WMH regions manually depicted by two observers was 0.878, which formed a solid basis for the development and evaluation of the automatic segmentation system. The proposed algorithm outperformed other state-of-the-art methods (uResNet, 3D V-Net and Visual Geometry Group network) in the segmentation of WMH and other coexisting intracranial lesions and was well validated on datasets with thick-slice magnetic resonance (MR) images and the 2017 medical image computing and computer assisted intervention WMH Segmentation Challenge dataset (with thin-slice MR images), all showing excellent effectiveness. Furthermore, our method can subclassify WMH to display the WMH distributions and is very lightweight. Additionally, in terms of correlation to visual rating scores, our algorithm showed excellent consistency with the manual delineations and was overall better than those from other competing methods. In conclusion, we developed an automatic WMH quantification framework for multiple application scenarios, exhibiting a promising future in clinical practice.

## Introduction

White matter hyperintensities (WMH) are common subcortical neuroimaging findings characterized by high signals in fluid-attenuated inversion recovery (FLAIR) and T2-weighted images (Wardlaw et al., 2013; Prins and Scheltens, 2015). With a high prevalence in senior individuals (more than 95%), WMH are primarily considered to be established markers of cerebral small vessel disease (CSVD) and are strongly correlated with an increased risk of cognitive impairment, ischemic stroke, and mood and gait disturbance (Simoni et al., 2012; Prins and Scheltens, 2015; Ter Telgte et al., 2018). In addition to its vascular etiology, WMH are also incidentally attributed to the pathology of other disorders, such as multiple sclerosis (MS), hypoxic-ischemic encephalopathy, and leukodystrophy. Hence, an accurate and reproducible method for WMH assessment is important. Manual delineation of the WMH by a neuroradiologist was thought to be a viable way to evaluate and quantify white matter abnormalities quantifiably. However, it is not only tedious and time consuming, but also has disadvantages such as large intra- and inter-observer variabilities, ranging from 10 to 68% (Grimaud et al., 1996; Zijdenbos et al., 2002; Styner et al., 2008), making it infeasible for clinical applications and large-scale studies.

In recent years, deep learning (DL) has attracted strong attention due to its powerful modeling capacities and the ability to automatically learn advanced features from data once given a task and has demonstrated great success in image classification (Krizhevsky et al., 2012), target detection (Ren et al., 2017), and image segmentation (Long et al., 2015). Studies have also focused on WMH segmentation using DL methods, including convolutional neural networks (CNNs; Ghafoorian et al., 2017), the lesion prediction algorithm (Schmidt, 2017), and U-Net (Li et al., 2018). In addition, Guerrero et al. (2018) introduced residual units into U-Net (uResNet) to reduce model complexity and accelerate convergence. Although many algorithms have been proposed for WMH segmentation, few could be applied for clinical use for the following reasons. First, most algorithms proposed previously were developed based on data with both 3D T1-weighted and FLAIR images of a thin thickness (1–3 mm) using 3D networks (Ghafoorian et al., 2017; Valverde et al., 2017; Li et al., 2018; Moeskops et al., 2018; Kuijf et al., 2019). Nevertheless, in both clinical practice and recent important cohort studies focused on CSVD (van Norden et al., 2011; Lawrence et al., 2013; Lambert et al., 2016; Su et al., 2017, 2018; Tay et al., 2020), magnetic resonance (MR) images were more frequently acquired based on thick-slice protocols (2D FLAIR images with a layer thickness of approximately 5 mm), according to the authorized recommendation for CSVD (STandards for ReportIng Vascular changes on nEuroimaging, STRIVE; Wardlaw et al., 2013). Furthermore, although 3D T1-weighted and FLAIR MRI with scanning protocols of thin-slice layers have been widely applied in some developed countries, 2D MRI scans with thick-slice layers are still most commonly performed in developing countries. Evidence suggests that 3D convolutions are less intuitive and may perform poorly (Abulnaga and Rubin, 2018) on 2D data, especially for WMH segmentation on 2D MR images with thick-slice protocols (Dadar et al., 2017). Second, the data of former studies (Gibson et al., 2010; Klöppel et al., 2011; Schmidt et al., 2012; Brosch et al., 2016; Griffanti et al., 2016; Bowles et al., 2017; Ghafoorian et al., 2017; Schmidt, 2017; Valverde et al., 2017; Guerrero et al., 2018; Li et al., 2018; Moeskops et al., 2018; Park et al., 2018; Kuijf et al., 2019) contain relatively small sample sizes (20∼200) limited to some specific groups, such as patients with CSVD and MS. Third, WMH usually coexist and coalesce with other types of brain lesions that have decided clinical values and sometimes also appear hyperintense on FLAIR or T2-weighted images, such as lacunes and large stroke lesions (Gouw et al., 2011), and these lesions were rarely identified and segmented simultaneously in prior works (Wang et al., 2012; Guerrero et al., 2018). Finally, the partial volume effect is stronger in 2D MR data with thick layer thickness for routine clinical practice and poses great challenges both in data labeling and algorithm development. Thus, the previously reported algorithms that were developed based on a single FLAIR sequence may also not be suitable for clinical practice.

**FIGURE 1:**
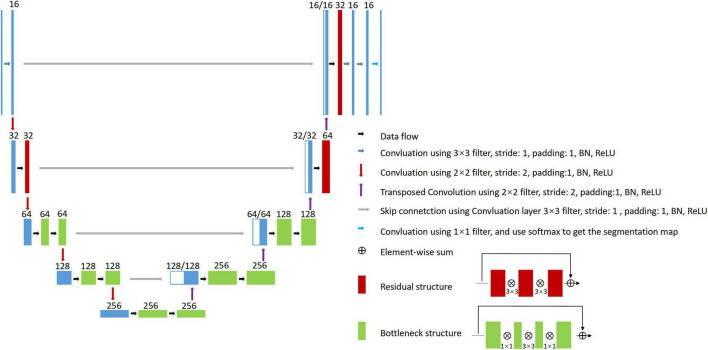
Proposed 2D VB-Net architecture for WMH segmentation.

To address the above issues, in this study, a large MR dataset of 1,045 WMH subjects was collected; these subjects had WMH of any degree and exhibited a wide range of demographic characteristics. The MR images were acquired by seven different scanners (3 T or 1.5 T), and each subject had T1-weighted, T2-weighted, and FLAIR images. We first proposed a series of semi-automated labeling protocols for manually labeling WMH and other intracranial lesions based on multi-modality images. We then presented a novel 2D CNN named 2D VB-Net (Figure 1) that incorporates the advantages of an efficient encoder-decoder framework for feature embedding, residual connections for information flow, and bottleneck layers for model compression. To validate the robustness and generalization of our algorithm, thorough experiments were performed on two independent in-house datasets (with thick-slice MR layers) and on the dataset from the 2017 medical image computing and computer assisted intervention (MICCAI) WMH Segmentation Challenge (with thin-slice MR layers of 3 mm). Furthermore, direct comparisons were performed between our method and three well-established CNN algorithms, uResNet (Guerrero et al., 2018), 3D V-Net (Milletari et al., 2016; Casamitjana et al., 2017), and the Visual Geometry Group network (VGGNet; Xu et al., 2018), in the segmentation of WMH and other intracranial lesions. Comparisons were also conducted between the correlations of the total and subclassified WMH volumes derived from automatic algorithms (our algorithm or the other methods) and manual raters with corresponding visual rating scores.

## Materials and methods

### Subjects

Magnetic resonance data were obtained from 1,045 subjects with WMH of any degree recruited from the outpatient and inpatient departments of Tongji Hospital, Wuhan, China. The exclusion criteria were as follows: (1) age less than 18 years old; (2) an intracranial space-occupying lesion or adults with intracranial surgery; (3) poor image quality due to significant movement artifacts or other factors; and (4) a lack of images from any of the three sequences (T1-weighted, T2-weighted, and FLAIR). The study was approved by the Ethics Committee of Tongji Hospital, Tongji Medical College, Huazhong University of Science and Technology. Written informed consent was obtained from each participant. The research was performed in accordance with relevant guidelines and regulations.

### Data preprocessing

The images were preprocessed according to the flowchart in Supplementary Figure 1. The steps were as follows:

a) The N4 algorithm (Tustison et al., 2010) was applied to T1-weighted images for bias field correction to handle the inhomogeneity of the magnetic field.
b) To address movements during scans, Advanced Normalization Tools (Avants et al., 2008) were used for multi-model registration. Because WMH are viewed most clearly on the FLAIR sequence, T1-weighted and T2-weighted images were registered to the FLAIR images *via* rigid and affine transformations computed based on the maximization of mutual information.
c) To focus network training on brain tissues, the FSL-Brain Extraction Tool (Smith, 2002) was applied to remove the skull from the T1-weighted images, and the corresponding skull regions were removed from the already registered T2-weighted and FLAIR images.
d) To minimize the variations in intensity ranges among scanners, subjects, and modalities and to boost network training convergence, quantile normalization was applied to all images to normalize the voxel intensities to the range of [0,1] using the equation:

(1) $$I^{{}^{\prime }}=\left\{ \begin{array}{c} 0\,\text{if}\,I<P_{0.001};\\ 1\,\text{if}\,I>P_{0.999};\\ \frac{I-P_{0.001}}{P_{0.999}-P_{0.001}}\,\text{otherwise}. \end{array}\right.$$

where *I* and *I’* are the original and normalized intensities, respectively. The original intensities were sorted, and the intensities at 0.1% and 99.9% were marked as *P*_*0.001*_ and *P*_*0.999*_, respectively.

### Proposed image delineation protocol

#### Multi-modality labeling

A single FLAIR sequence may not well differentiate WMH from lacunes, perivascular space (PVS), cortical infarctions, and other brain lesions (Wardlaw et al., 2013), especially in 2D MR data with thick slices in which the partial volume effect is even stronger. In this work, we cross-checked all three sequences for the delineation of WMH regions and other intracranial lesions, which were assigned as label 1 and label 2, respectively.

#### Use of semi-automated tools to reduce manual error

Manual delineation of WMH is a challenging task (García-Lorenzo et al., 2013). Cognitive differences and manual errors of boundary delineation were the main reasons for large inter-observer variations and low Dice consistency. To reduce the inherent error of manual delineation and improve labeling consistency, a histogram-based threshold was used to extract the lesion from the FLAIR images, and experts then corrected the results on the registered T1-weighted and T2-weighted images using ITK-SNAP (Yushkevich et al., 2006).

#### Discussion to reduce cognitive differences

Two skilled observers independently delineated WMH regions guided by a neuroradiologist with 15 years of experience (S.B.X.). For any set of images, if the Dice value of two WMH delineations was lower than 0.5, the two observers discussed the case and then performed the delineation again independently. This process was repeated until the Dice value was consistently above 0.5. The Pearson’s correlation coefficient (PCC) was applied to evaluate the consistency between the two delineations.

#### Handling inter-observer ambiguity

Supplementary Figures 6D,E show the WMH regions on a sample of FLAIR images labeled independently by the two observers. The two sets of labels overlapped and exhibited discrepancies at times. Supplementary Figure 6F shows the union of the two delineations, and these results were considered the ground truth. To handle inter-observer variations, we proposed the following labeling definition. As shown in Supplementary Figure 6G, the intersections of the two sets of labels (marked red) were defined as the “definite” WMH, while the regions labeled by only one of the observers (marked blue) were defined as the “suspected” WMH. The union of the two sets was regarded as the ground truth for training, but the voxels in the definite and suspected WMH regions were given unequal weights.

#### Silver standard dataset

In clinical images, no real ground truth is available (García-Lorenzo et al., 2013), especially in WMH segmentation, and the best approximation is manual delineation by experts. However, inter-observer variation is unavoidable, and a silver standard dataset is needed to evaluate the accuracy of segmentation. In the present study, 20 out of the 1,045 subjects were selected to form the silver standard dataset, covering subjects with light to severe WMH loads. The images of each of the 20 subjects were labeled using the method described previously by five neuroradiologists with over 8 years of experience independently (XL, WZ, HH, SX, and WQ). The five sets of labeled results were merged by majority voting on every labeled voxel. The remaining 1,025 subjects were labeled by two experienced observers using the labeling strategy described earlier.

### Proposed 2D VB-Net architecture

We previously proposed a VB-Net framework for 3D organ segmentation on CT images. It was ranked first in the Segmentation of Thoracic Organs at Risk in CT images challenge held by the IEEE International Symposium on Biomedical Imaging 2019 (Han et al., 2019). Here, we adopted this framework and customized it into a network structure named 2D VB-Net with weighted Dice loss to handle the WMH segmentation problem.

Briefly, V-Net (Milletari et al., 2016) is used as the backbone of the network, which includes the encoder, decoder, and residual blocks. The bottleneck layer was introduced to reduce the number of feature maps. The input to the network combined the T1-weighted, T2-weighted, and FLAIR images because clinically these three MRI modalities were jointly recommended to determine the territory of WMH and differentiate WMH from other cerebral lesions, such as lacunes, PVS and large infarctions. The adjustments of the network are listed as follows.

1. 2D convolutional kernels replace 3D kernels since the MR images were acquired using the 2D protocol with a layer thickness of no less than 5 mm and the number of slices in a series was usually 16∼20.
2. The number of bottleneck structures of the high levels was reduced. Here, we referred to the vertical depth as the level, with the original input and output depth as level 1; after down-sampling 4 times as level 5, the lower the level was, the higher the spatial resolution was but with fewer feature maps, and vice versa. Because the number of segmentation categories is relatively small, reduction of the bottleneck structure would not impact the segmentation accuracy but would help reduce the model parameters and hence improve the robustness.
3. The output block was adjusted, and two convolutional layers were added to the original basis to generate the segmentation probability map.

The architecture of the 2D VB-Net is shown in Figure 1. In general, the left side of the 2D VB-Net was the contraction path, and the right side was the expansion path. The left contraction path reduces the size of input by down-sampling. It consists of four blocks, which comprise two convolution layers, bottleneck structures and residual structures. The right expansion path has similar structures and recovers the semantic segmentation image by de-convolutions. Between the two paths, a skip connection was introduced to improve the segmentations. The numbers of input channels and output channels are not shown in Figure 1, as these were configurable. In this study, there were 3 input (T1-weighted, T2-weighted, and FLAIR) and 3 output (background, WMH, other intracranial lesions) channels and the input crop size was 256 × 256, the parameters of 2D VB-Net were only 1.08 M, which accounted for only 3.5% of that of U-Net. Benefit from introducing the bottleneck structures, the inference memory was 696 M. An extra benefit of the lightweight 2D VB-Net would be its ease of deployment for a cloud or mobile application.

Next, we introduced weighted Dice loss to focus on definite WMH. WMH regions were very sparse compared to background regions, causing an imbalance between positive and negative samples. This situation was also common in medical image segmentation problems. Hence, the Dice loss (Milletari et al., 2016) was used to avoid local minima in the training process based on the following equation:


(2) $$\mathrm{Dice}\,\mathrm{loss}=1-\,\frac{1}{C}\,\sum_{1}^{C}\left(\frac{2\,\sum_{i}^{N}p_{i}^{c}\,g_{i}^{c}}{\sum_{i}^{N}{\left(p_{i}^{c}\right)}^{2}+\sum_{i}^{N}{\left(g_{i}^{c}\right)}^{2}}\right)$$

where *p*_*i*_ ∈ *P* is the algorithm result, *g*_*i*_ ∈ G is the ground truth, and *C* is the number of classes. As described previously, we defined definite WMH and suspected WMH. Regarding Dice loss, more weight was placed on the definite WMH, and therefore the Dice loss was modified as


(3) $$\mathrm{Dice}\,\mathrm{loss}=1-\,\frac{1}{C}\,\sum_{1}^{C}{\left(w\,\left(x\right)\right)}^{c}\,\left(\frac{2\,\sum_{i}^{N}p_{i}^{c}\,g_{i}^{c}}{\sum_{i}^{N}{\left(p_{i}^{c}\right)}^{2}+\sum_{i}^{N}{\left(g_{i}^{c}\right)}^{2}}\right)$$

where ^(*w*(*x*))*c*^ is the weight map.

For definite WMH, the weight was set to 2, while the weight for the suspected WMH was set to 1 + *f*(dis(Def)), where dis(Def) was the distance to the center of the nearest definite WMH region. The *f*(dis(Def)) ranged from 0 to 1 and was calculated as


(4) $$f\,\left(\text{dis}\,\left(\text{Def}\right)\right)=1-\frac{\text{dis}\,\left(\text{Def}\right)}{\text{dis}\,{\left(\text{Def}\right)}_{\text{max}}}$$

### Evaluation metrics

Dice, *recall*, and *precision* as common indictors for quality image segmentation were also used in this study. Specifically, Dice described the overlap between the segmentation regions and the ground truth regions as


(5) $$\text{Dice}=\frac{2*T\,P}{2*T\,P+F\,P+F\,N}.$$

and *recall* described the portion of ground truth voxels that were corrected segmented as


(6) $$r\,e\,c\,a\,l\,l=\frac{T\,P}{T\,P+F\,N}$$

*Recall* was computed for all the labeled WMH regions combined, as well as separately for the definite WMH regions. *Precision* described the portion of segmented voxels that belong to the ground truth regions as


(7) $$p\,r\,e\,c\,i\,s\,i\,o\,n=\frac{T\,P}{T\,P+F\,P}$$

In the above equations, *TP*, *FN*, and *FP* denote the number of true positive, false negative and false positive voxels, respectively.

In addition to voxel-level *recall* and *precision*, lesion-level *recall* and the F1-score were also defined as performance evaluation metrics. Each lesion was defined as a 3D connected component, and the formula for *recall* for each lesion was the same as in equation (Styner et al., 2008), but *TP* and *FN* were redefined as the number of lesions that were correctly segmented or missed by the algorithm. The F1-score was the harmonic mean of *recall* and *precision* and was calculated as


(8) $$\text{F}\,1=\frac{2*r\,e\,c\,a\,l\,l*p\,r\,e\,c\,i\,s\,i\,o\,n}{r\,e\,c\,a\,l\,l+p\,r\,e\,c\,i\,s\,i\,o\,n}$$

To better distinguish *recall* and *precision* parameters at the voxel level, the lesion-level *recall* and F1-score were named lesion recall and lesion F1, respectively. Additionally, the average volume difference (*AVD*, in percentage) was defined as


(9) $$A\,V\,D=\frac{\left|A-B\right|}{B}*100\%$$

The *Hausdorff* distance (95th percentile): *Hausdorff95* was also included, which indicated the distances of the two lesion voxel sets with a percentile value of 95% and was calculated as


$$Hausforff95=\text{max}\{\max{\left(95\%\right)}_{a\in A}{\text{min}}_{b\in B}||a-b||,$$

(10) $$\max{\left(95\%\right)}_{b\in B}{\text{min}}_{a\in A}||b-a||\}$$

where *A* is the segmented image and *B* is the ground truth image, which denotes the absolute difference in percentage. In the above, *TP, FN*, and *FP* denoted the number of true positive, false negative and false positive voxels, respectively.

### Subclassification of white matter hyperintensities

On the basis of traditional classification, which divided WMH into periventricular WMH (PWMH) and deep WMH (DWMH; Fazekas et al., 1987), literature further proposed subclassifying WMH into 4 categories based on their distance from the ventricle, including juxtaventricular WMH (JVWMH), PWMH, DWMH, and juxtacortical WMH (JCWMH; Kim et al., 2008; Li et al., 2019). Briefly, JVWMH is defined as the area within 3 mm from the ventricle, and PWMH ranges from 3 mm to 13 mm; JCWMH refers to WMH within 4 mm from the corticomedullary junction, and DWMH is defined as the region between PWMH and JCWMH (Kim et al., 2008). In our system, we provided such subclassification of WMH, and thus, their specific volumes could be obtained as extra features. To accomplish this analysis, we first segmented the ventricles from MR images by using the 2D VB-Net structure and then constructed a distance map where voxels in the ventricle have a distance of 0 and all other brain voxels are assigned values as their nearest distances from any ventricle voxel. The whole-brain WMH was thus subclassified into these 4 categories based on the distance map. In addition, we also subclassified WMH into only 2 categories in line with the traditional classification (PWMH, defined as WMH within 10 mm of the lateral ventricle, and other WMH defined as DWMH; DeCarli et al., 2005; Godin et al., 2010; Lampe et al., 2019; Sundaresan et al., 2019) based on the criteria proposed by Fazekas et al., and showed the segmentation maps.

### Correlation analysis with the visual rating score

Furthermore, to investigate the clinical value of these WMH volume features proposed in this study, we evaluated their correlations with the Fazekas visual rating scale, the most widely used visual inspection approach for WMH assessment in the combined two independent datasets (Fazekas et al., 1987). The total Fazekas score (0–6) is defined as the sum of the PWMH score (0–3) and the DWMH score (0–3; Fazekas et al., 1987; Helenius and Henninger, 2015). For each subject, the Fazekas scores of PWMH, DWMH, and the total Fazekas score were obtained from the consensus from two experienced neuroradiologists (WZ and HH) who were blinded to the clinical information and the segmentation maps.

To clinically validate our method, we further evaluated the correlations between WMH volumes (extracted with our system or manual raters) and the Fazekas scale. The statistical analysis was performed in R version 4.1.1 (http://www.R-project.org)^1^. Correlation analysis was conducted between the volumes of the total WMH, PWMH or DWMH (expressed as a percentage of the total intracranial volume) and the corresponding Fazekas score using Spearman’s correlation. To verify whether the proposed algorithm is a valid alternative method to manual annotations and whether the algorithm is better than other competing methods in clinical correlation analysis, we compared the correlation coefficients between Fazekas scores and WMH volumes generated by 2D VB-Net or other state-of-the-art algorithms and the correlation coefficients between Fazekas scores and WMH volumes generated by experienced manual raters assisted with semi-automatic tools (used as the ground truth for the algorithm training). A bootstrap approach was used to estimate the 95% confidence interval (CI) of the correlation (*N* = 1,000), and a significant difference between two Spearman’s correlation coefficients was defined by a 95% CI that did not include 0 (Efron, 1987; Wood, 2004). Additionally, we also repeated the analyses above using the extracted PWMH and DWMH according to the widely used definition mentioned above.

## Results

The in-house dataset included the image data of 1,045 subjects using seven types of MRI scanners, including GE Signa HDxt 3.0 T, GE Discovery MR750 3.0 T, UIH uMR 780 3.0 T, GE Signa Excite 1.5 T, GE Signa HDxt 1.5 T, GE Signa Creator 1.5 T, and GE Brivo MR355 1.5 T. The scanning protocols were all 2D axial, and the details of the protocols and the subjects’ distribution over imaging devices are listed in Supplementary Tables 1, 2. Supplementary Figure 2 shows the data used in this work from different views and modalities. The dataset was divided into the following subsets for algorithm testing and validation: (1) the multi-scanner set (849 subjects with data from both 3 T and 1.5 T MR scanners); (2) the independent dataset 1 (IDS 1, 102 subjects with data acquired from a 3 T MR scanner); (3) the independent dataset 2 (IDS 2, 74 subjects with data acquired from a 1.5 T MR scanner); and (4) the silver standard dataset (20 subjects randomly chosen from both 3 T and 1.5 T MR scanners). The demographic characteristics of the subjects in each dataset are summarized in Table 1.

**TABLE 1:** Demographic features of the in-house datasets.

	Multi-scanner dataset	IDS 1	IDS 2	Silver standard dataset	Total
	(*N* = 869)	(*N* = 102)	(*N* = 74)	(*N* = 20)	(*N* = 1045)
Age (Years)	62.19 ± 11.33	64.72 ± 9.71	60.19 ± 11.50	62.30 ± 9.97	62.30 ± 11.20
Gender (M/F)	457/392	51/51	36/38	15/5	559/486

IDS, independent dataset.

### Image delineation

**FIGURE 2:**
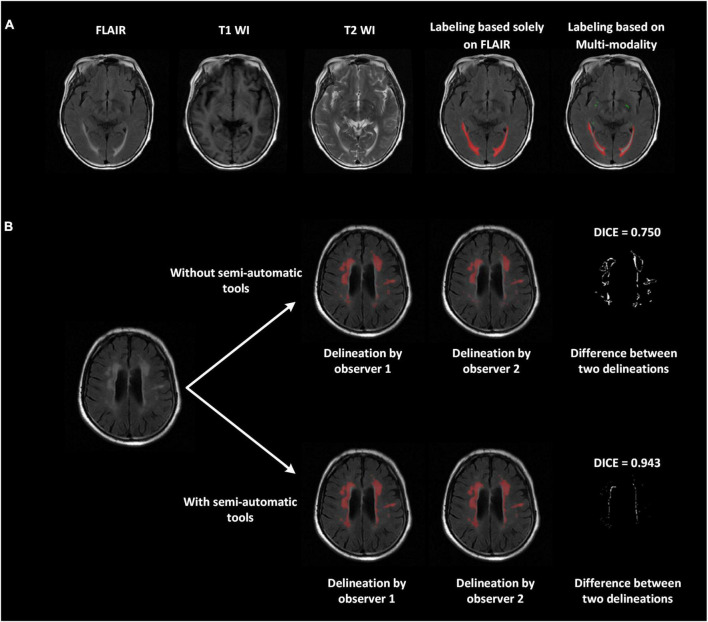
Improved delineation with our labeling strategies. **(A)** Multi-modality images (T1-weighted, T2-weighted, and FLAIR) applied to differentiate WMH and other intracranial lesions and reduce false positives. The ventricular regions were correctly avoided. The perivascular space, marked in green, was also labeled jointly on all three modalities. **(B)** With the use of semi-automatic tools, the difference between the two delineations declined, and the Dice improved from 0.75 to 0.943.

We proposed a series of advanced labeling strategies. The multi-modality images were used to differentiate WMH and other intracranial lesions and were effective in reducing the false-positive parts of WMH regions (ventricular regions were correctly avoided in this example) and improving the accuracy of labeling other intracranial lesions (PVSs in this case; Figure 2A). As shown in Figure 2B, the strategy of using semi-automatic tools was utilized for manual delineation to reduce manual errors of boundary delineation and the Dice consistency was significantly improved with the help of semi-automatic tools. Compared to the completely manual method, using our labeling strategies, the Dice consistency significantly improved from 0.538 to 0.878, and the PCC improved from 0.963 to 0.993 for WMH delineation in 280 randomly chosen subjects. For other intracranial lesions, the Dice consistency significantly improved from 0.475 to 0.602, and the PCC improved from 0.955 to 0.992 (*P* < 0.01). We have released a portion of delineation data at https://github.com/haohaohuang-tj/WMH.

### 2D VB-Net

The proposed 2D VB-Net fully convolutional network (FCN; Figure 1) was implemented in the PyTorch DL framework, with weighted Dice loss as the loss function. To handle the variations in pixel spacing among scanners, the images and labeled regions were uniformly resampled in both the *x*- and *y*-directions to a spacing of 0.5. The pixel spacing in the *z*-direction remained intact. The input 2D patches for training were 256 × 256 and randomly sampled from the image volume. The initialization of weights used the Kaiming initialization strategy (He et al., 2015), and the optimizer was adaptive moments estimation (Adam; Kingma and Ba, 2014) with the first moment coefficient = 0.9, second moment coefficient = 0.999, learning rate = 0.001, and mini-batch size = 48. The training was carried out on 2 NVIDIA Titan Xp 12 GB graphics processing units. In addition, we added a detailed network layer description table to facilitate the readers’ performance (Supplementary Table 3). The code and pre-trained model are publicly available at https://github.com/simonsf/wmh-segmentation.

### Five-fold cross-evaluation on the multi-scanner dataset

**FIGURE 3:**
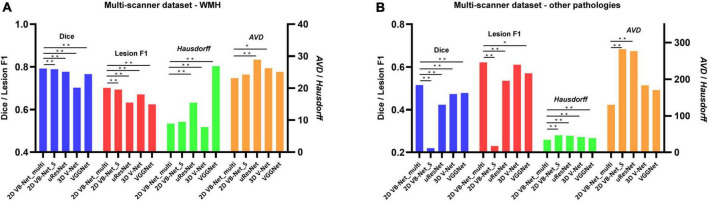
Performance differences among the algorithms on the multi-scanner dataset. **(A)** Differences in the performance of WMH segmentation among the five methods; **(B)** Differences in the segmentation performance of other intracranial pathologies among the five methods. The performance of each algorithm was measured by Dice, lesion F1, *Hausdorff*, and *AVD*, respectively. Of note, the 2D VB Net_multi, uResNet, 3D V-Net, and VGGNet were trained and evaluated based on multi-modality MR images (T1-weighted, T2-weighted, and FLAIR), whereas the 2D VB Net_S was trained and evaluated based on only FLAIR images. A lower value of *Hausdorff* and *AVD* indicates better performance of the algorithm. *, ** *P*-value of two-tailed paired *t*-test between the performance of the 2D VB-Net _multi and the other algorithms; *, *P* < 0.05 significance level; **, *P* < 0.001 significance level.

**FIGURE 4:**
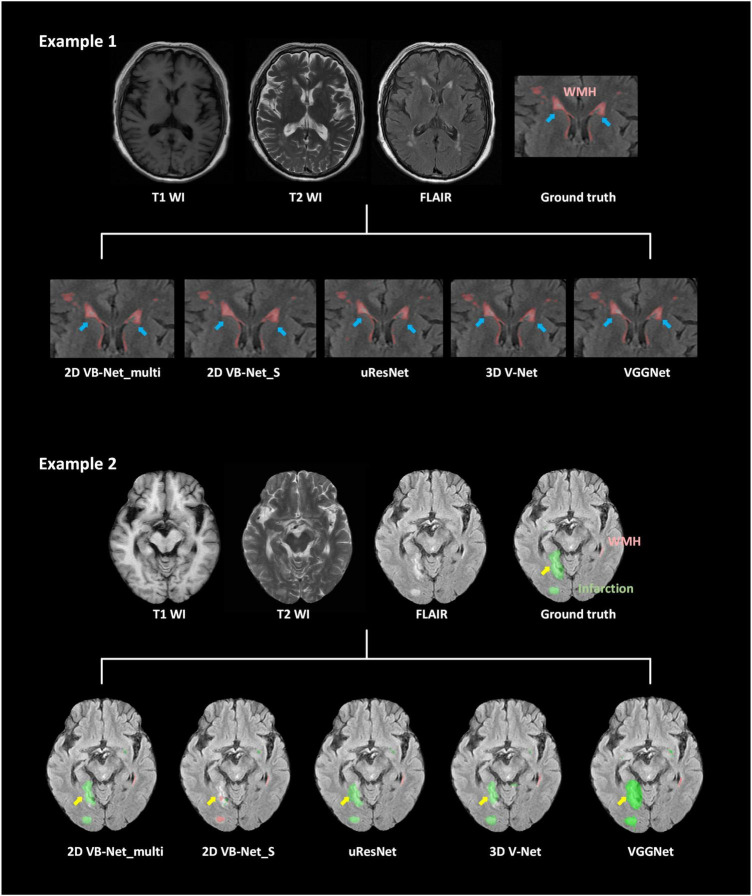
Examples of different segmentation methods used to illustrate the segmentation of WMH and cerebral infarction. The blue arrows show hyperintensities adjacent to the bilateral anterior horn of lateral ventricles on the FLAIR sequence. A portion of these hyperintensities is located in the lateral ventricles because of the partial volume effect, which was misidentified as WMH by using the 2D VB-Net_S algorithm. The yellow arrows indicate cerebral infarctions located in the brain areas of the cortex and cortical-subcortical junction. The 2D VB-Net_S algorithm misidentified these lesions as WMH. All the other algorithms correctly identified the stroke lesions, and the 2D VB-Net_multi algorithm provided better segmentation than the other three state-of-the-art algorithms.

The results of five-fold cross-validation of the proposed multi-modality algorithm (noted as 2D VB-Net_multi), FLAIR-only algorithm (noted as 2D VB-Net_S), uResNet, 3D V-Net, and VGGNet (note: these three algorithms for comparison were all trained using multi-modality MR images), are shown in Figure 3 and Supplementary Table 4. Overall, the 2D VB-Net_multi algorithm achieved the best results among the five algorithms, as measured by Dice, *Hausdorff* and lesion F1 measurements, indicating the great effectiveness of the 2D VB-Net algorithm on the segmentation of WMH and coexisting intracranial lesions. Two examples of segmentation maps using the different methods are shown in Figure 4. which demonstrates that the single-modality algorithm (2D VB-Net_S) misidentified some regions (a portion of anterior horns of the lateral ventricles in example 1 and cerebral infarction located in brain areas of the cortex and cortical-subcortical junction in example 2) showing hyperintensity on the FLAIR sequence as WMH. Furthermore, with reference to the manual delineations, the 2D VB-Net_multi algorithm overall segmented WMH and other intracranial lesions better than the competitive methods.

### Validation on two independent datasets

**FIGURE 5:**
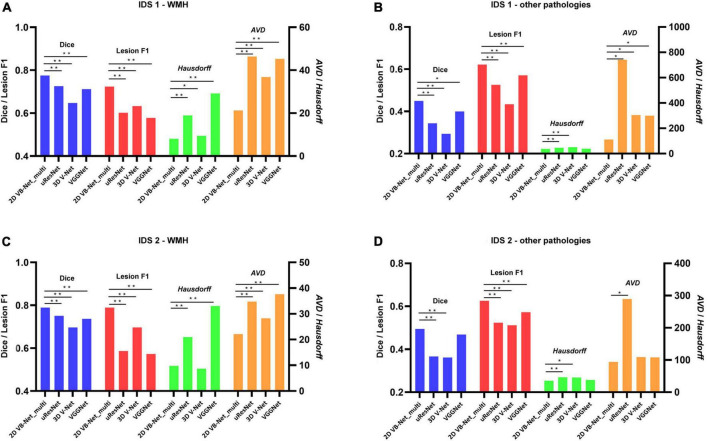
Performance differences between 2D VB-Net and the other state-of-the-art algorithms on the two independent in-house datasets. The upper row shows the differences in segmentation performance in WMH **(A)** and other intracranial pathologies **(B)** among the four methods on IDS 1; **(B)** the lower row shows the differences in segmentation performance of WMH **(C)** and other pathologies **(D)** among the four methods on IDS 2. The performance of each algorithm was measured by Dice, lesion F1, *Hausdorff*, and *AVD*. Of note, the four algorithms were trained and evaluated based on multi-modality MR images (T1-weighted, T2-weighted, and FLAIR), and a lower value of *Hausdorff* and *AVD* indicates better performance of the algorithm. IDS, independent dataset. *, ** *P*-value of two-tailed paired *t*-test between the performance of 2D VB-Net _multi and the other algorithms; *, *P* < 0.05 significance level; **, *P* < 0.001 significance level.

To further evaluate the generalization capability of the multi-modality algorithms, the 2D VB-Net and the other 3 state-of-the-art algorithms were trained on IDS 1 and IDS 2, respectively. Here, the 2D VB-Net also achieved fairly good performance and outperformed the other three well-established algorithms, showing fewer performance drops compared to the other algorithms (Figure 5 and Supplementary Table 5). Furthermore, we subdivided the samples in IDS 1&2 into three groups with different WMH volumes of <5 ml, 5–15 ml, and >15 ml. Generally, performance was demonstrably higher on larger lesions, which was also the case for human delineations. On both independent datasets, the 2D VB-Net algorithm showed significantly better performance in all three groups compared to that of the other three competitive algorithms as measured by Dice except in the group with severe WMH (WMH volume > 15 ml) of IDS 2 compared to that of uResNet and VGGNet (Supplementary Table 6).

### Validation on the medical image computing and computer assisted intervention white matter hyperintensities challenge dataset

The algorithms were also evaluated using the 2017 MICCAI WMH Segmentation Challenge dataset with a 5-fold cross-evaluation. As listed in Supplementary Table 7, the dataset consisted of 60 subjects from 3 hospitals, and each case contained FLAIR and T1-weighted images. The public data had been subjected to field correction and were already registered. Skull stripping and intensity normalization were conducted on these data in the same way as that during the preprocessing step regarding our data. The data were then resampled in the axial direction to a resolution of 1 mm, and the input patch was adjusted to 128 × 128. The performance of the top 4 algorithms (Kuijf et al., 2019) in the 2017 MICCAI WMH Segmentation Challenge is listed in Table 2. The proposed 2D VB-Net algorithm also showed good performance (Dice = 0.789, lesion F1 = 0.764) on this small dataset with thin-slice MR images.

**TABLE 2:** Performance of the algorithms for WMH segmentation on the challenge public dataset.

Team	Method	Dice	*Recall*	*Precision*	*Hausdorff*↓	*AVD*↓	Lesion recall	Lesion F1
Our method	2D VB-Net	0.789	0.792	0.805	4.946	18.106	0.807	0.764
sysu media (Kuijf et al., 2019)	CNN	0.80	–	–	6.30	21.88	0.84	0.76
cian (Kuijf et al., 2019)	MDGRU	0.78	–	–	6.82	21.72	0.83	0.70
nlp logix (Kuijf et al., 2019)	CNN	0.77	–	–	7.16	18.37	0.73	0.78
nic-vicorob (Kuijf et al., 2019)	CNN	0.77	–	–	8.28	28.54	0.75	0.71

The table shows the performance of our method and the algorithms ranked in the top 4 for WMH segmentation on the 2017 MICCAI WMH Segmentation Challenge public dataset, as reported by Kuijf et al. (2019). Lower Hausdorff and AVD values indicate better performance of the algorithm.

### Subclassification of white matter hyperintensities and correlation analysis with the visual rating score

**FIGURE 6:**
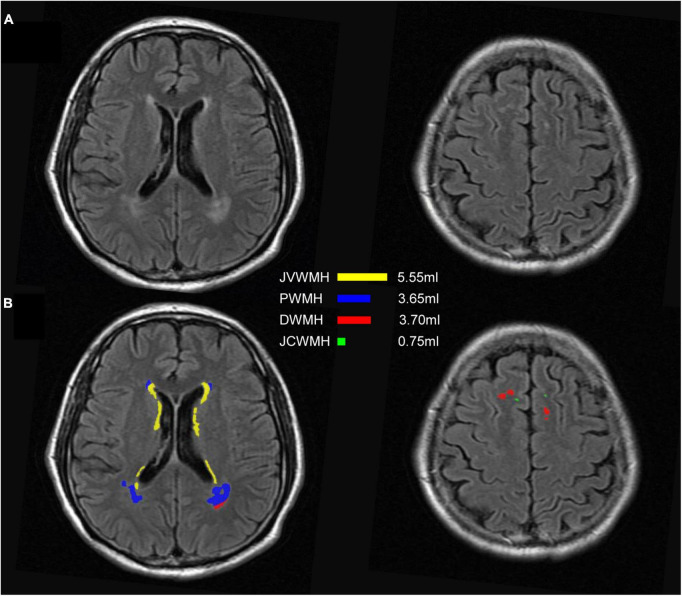
An example of segmentation results for subclassified WMH according to Kim’s criteria. **(A)** FLAIR images; **(B)** display of the segmentation map and the volumes of each subclassified WMH area according to Kim’s criteria. The regions of each subclassified WMH are represented by different colors. DWMH, deep white matter hyperintensities; JVWMH, juxtaventricular white matter hyperintensities; JCWMH, juxtacortical white matter hyperintensities; and PWMH, periventricular white matter hyperintensities.

As shown in Figure 6, the WMH was divided into four parts: the JVWMH, the PWMH, the DWMH, and the JCWMH. Each subclassification of WMH is labeled in a different color, and the volume of each area is also displayed. Furthermore, we calculated the correlations between the volume of WMH obtained by manual delineation or the automatic methods and the Fazekas scores. In terms of correlation to Fazekas scores, our algorithm showed excellent consistency with the manual delineations, and compared to other competing methods, the correlation coefficients using volumes generated by our algorithm showed overall better consistency with the coefficients using volumes derived from manual raters, especially for DWMH (Table 3). In addition, when WMH were only subdivided into two parts (PWMH and DWMH) based on the criteria proposed by Fazekas et al. (1987), the results of the correlation analysis using the volumes of PWMH and DWMH according to the definitions widely used in previous studies (within or outside 10 mm from the lateral ventricle; DeCarli et al., 2005; Godin et al., 2010; Seo et al., 2012; Griffanti et al., 2018; Lampe et al., 2019) were very similar to the results of the correlation analysis using the volumes of PWMH and DWMH based on Kim’s criteria (Kim et al., 2008; Supplementary Figure 3 and Supplementary Table 8).

**TABLE 3:** Correlation analysis of WMH volumes based on Kim’s criteria extracted from manual delineation and algorithms with Fazekas scores and comparisons between correlation coefficients.

	Total WMH			PWMH			DWMH		
	*r*	Δ*r*	95% Bootstrap CI of Δ*r*	*r*	Δ*r*	95% Bootstrap CI of Δ*r*	*r*	Δ*r*	95% Bootstrap CI of Δ*r*
Manual delineation	0.895			0.870			0.850		
2D VB-Net	0.900	–0.005	(–0.0251, 0.0132)	0.879	–0.009	(–0.0227, 0.0013)	0.834	0.016	(–0.0010, 0.0356)
uResNet	0.890	0.005	(–0.0221, 0.0309)	0.864	0.006	(–0.0133, 0.0340)	0.817	**0.033**	**(0.0035, 0.0774)**
3D V-Net	0.872	0.023	(–0.0043, 0.0571)	0.857	0.013	(–0.0079, 0.0398)	0.802	**0.048**	**(0.0101, 0.0999)**
VGGNet	0.868	0.027	(–0.0003, 0.0678)	0.865	0.005	(–0.0131, 0.0296)	0.793	**0.057**	**(0.0218, 0.1187)**

r refers to Spearman’s correlation coefficient of WMH volumes extracted from manual delineations or algorithms with corresponding Fazekas visual rating scores. Δr refers to the difference between Spearman’s correlation coefficient of manual-annotated WMH volumes with Fazekas scores and Spearman’s correlation coefficient of WMH volumes extracted from each automatic algorithm with Fazekas scores, using a Bootstrap method for 1,000 bootstrapping times. Bold values represent a significance of Δr that is defined by a 95% Bootstrap CI entirely above or below 0 (uncorrected, considering that the comparative analysis is exploratory). CI, confidence interval; DWMH, deep white matter hyperintensities; and PWMH, periventricular white matter hyperintensities.

### Evaluation on the silver standard dataset

2D VB-Net was evaluated on the silver standard dataset, which was labeled by 5 neuroradiologists with over 8 years of experience and merged by majority voting. The network was first re-trained on all the data in the multi-scanner set and the two independent datasets and then tested on the silver standard dataset. The algorithm again demonstrated its effectiveness in WMH segmentation, delivering Dice, *Hausdorff*, *AVD* and lesion recall values of 0.782, 10.266, 44,635, and 0.853, respectively (Supplementary Table 9). Segmentation performance was less accurate for other intracranial lesions than for WMH, but lesion recall was as high as 0.908, indicating its good capacity in lesion detection. For more detailed observation, the WMH load of each case and the Dice value achieved by the algorithm are listed in Supplementary Table 10. The best case and the worst case are also shown in Supplementary Figures 4, 5. Furthermore, we conducted a direct comparison of the performance of the manual delineations using semi-automatic tools by an experienced observer independently and the proposed fully automated method in the silver standard dataset since the delineation results by using the two methods could be compared to the “silver standard.” The results showed that our proposed fully automated method achieved overall better performance on the task of WMH segmentation compared to that of the semi-automatic method when the data were independently labeled by an experienced observer (see details in Supplementary Table 11).

## Discussion

In this study, a robust system for automated WMH segmentation in multiple application scenarios was proposed. A novel automated algorithm, 2D VB-Net FCN, was developed and evaluated for the segmentation of WMH based on multi-modal MR images in a large dataset of 1,045 subjects across various demographics. With a series of advanced labeling strategies, the 2D VB-Net algorithm, adopted from the VB-Net solution for 3D volume data, achieved higher accuracy regarding WMH segmentation of 2D MR images on data with various imaging protocols and clinical diagnoses compared to the accuracy of other state-of-the-art methods. Notably, this WMH segmentation system was constructed based on the 2D thick-slice protocol, which is more frequently applied for routine clinical acquisition, using multiple scanners and could suitably identify and segment other commonly coexisting intracranial lesions of vascular origin (i.e., lacunes, cortical infarcts, intracranial hemorrhage, and PVS). Furthermore, to display detailed information of the WMH distribution, the system can subclassify WMH into 4 categories and provide a visual interface to show the segmentation map and volumes of each subclassified WMH. In the validated analysis, the proposed method was well hick-svalidated on two independent datasets with tlice MR images and the 2017 MICCAI WMH Segmentation Challenge dataset with a thin layer slice of 3 mm. In addition, in terms of correlation to Fazekas scores, our algorithm showed excellent consistency with the manual delineations and overall correlated better with the visual rating scores than other competing methods, highlighting the robustness and clinical value of our algorithm.

The main strength of our study is that we proposed a clinically usable and competitive method for the automatic segmentation of WMH in multiple application scenarios, including WMH coexisting with other intracranial lesions and using MR images with various imaging protocols. Constructed based on the largest dataset to date with the improvement for the WMH segmentation, our algorithm can well segment WMH using MR images with both thin-slice and think-slice layers. Furthermore, with the multi-modal labeling and training protocols, other intracranial lesions of vascular origin usually coexisting with WMH were properly labeled and segmented by our method. Of note, some pathologies also appear hyperintense on the FLAIR sequence and are difficult to distinguish from WMH, such as large stroke lesions located in cortical-subcortical areas and lacunes without the suppression of the central cavity on the FLAIR sequence (Wardlaw et al., 2013). In most previous studies focused on WMH segmentation, either patients with other intracranial lesions have been excluded or these lesions were not labeled, and the accuracy of the algorithms in identifying other intracranial lesions was fairly low (Supplementary Table 12). In contrast, our algorithm could not only be applied for automatic WMH segmentation in various diseases, but could also show the extent of other brain lesions that often coexist with WMH intuitively and accurately, which may provide clinicians with more information and effective reminders to recognize subtle lesions and make a proper diagnosis.

Another highlight and strategy for improving the accuracy and generalization of our study is the proposed novel labeling pipeline. Dedicated labeling of WMH is difficult according to the obscure boundary of WMH, other coexisting lesions easily being confused with WMH, and the partial volume effect which is particularly strong in thick-slice MR images. For these reasons, the consistency of manual WMH labeling between two experienced observers was mediocre, especially for subjects with mild WMH load. In this study, we proposed a series of labeling strategies, including using semi-automatic tools to assist labeling, and labeling on multi-modality images to reduce the possibility of misjudging other lesions and normal intracranial structures as WMH, and discussion of largely discrepant regions followed by independent delineation helped reduce cognitive differences. After applying these rules during delineation, inter-observer consistency showed a dramatic improvement in the large dataset, which forms the basis for the development and evaluation of a solid automated segmentation system.

2D VB-Net is a new network structure proposed in this paper, adopted from 3D VB-Net (Han et al., 2019), with adjustments made to fit the task of WMH segmentation. VB-Net (Han et al., 2019) performed well in the segmentation of medical volume data, indicating that sampling 3D patches from images could obtain inter-slice information and provide better segmentation accuracy. In this study, the data we utilized for developing the algorithm were 2D thick-slice MR images from real-world clinical practice, leading to the relatively low relationship between consecutive slices. Because the number of slices was small, if a 3D network was used, resampling on the *Z*-axis was warranted to increase the number of slices, which would greatly increase the amount of calculation and bring accuracy error problems (Dadar et al., 2017; Abulnaga and Rubin, 2018). Hence, here, we used the 2D convolution kernel instead of the 3D convolution kernel. In the architecture of the network, the proposed 2D VB-Net made several modifications for improvement: (1) skip connections are added in each layer to integrate low- and high-level information; (2) bottleneck layers are added to reduce the size of feature maps and thus increase the network efficiency; (3) the loss function is redesigned to promote the Dice loss of the WMH region, and (4) appropriate weights are assigned for the regions where two manual raters agree. After these adjustments, compared to other standard fully supervised CNNs, the proposed method incorporated the advantages of an efficient encoder-decoder framework for feature embedding, residual connections for information flow, bottleneck layers for model compression and is more appropriate for automatic segmentation of WMH and other intracranial lesions, and the method is also more lightweight for WMH segmentation than other competitive methods, which would be easier to deploy for a cloud or mobile application (Supplementary Table 13).

A large number of experiments were conducted to evaluate the accuracy and generalization of 2D VB-Net. Three state-of-the-art algorithms, uResNet, 3D V-Net, and VGGNet, which have shown good performance in WMH segmentation, as reported (Casamitjana et al., 2017; Xu et al., 2018), were used as comparable algorithms in some of the experiments. To make a fair comparison, the spacing and patch size parameters were adjusted for all the algorithms to reach the best performance on our data. The performance seemed to improve as the patch size increased, but a large patch size meant more computing resources (Supplementary Table 14).

The experiments on the multi-scanner dataset and the independent datasets demonstrated superior performance of 2D VB-Net in the segmentation of WMH and other intracranial pathologies compared to the three competitive algorithms, and WMH segmentation using multi-modality images had better performance than the method using only a single FLAIR sequence (Caligiuri et al., 2015; Li et al., 2018), as verified in this report. The advantage was even more obvious for the segmentation of other intracranial pathologies. The experiments on the two independent datasets (IDS 1&2) resulted in slight performance drops compared to the five-fold cross-validation tests on the multi-scanner set, probably because the data were acquired with different scanners. However, the performance of the proposed 2D VB-Net was still satisfactory and manifested good generalization. It should be noted that even if compared to the manual delineations using semi-automatic tools by an experienced observer independently, our proposed fully automatic method achieved overall superior performance on the segmentation of WMH (Supplementary Table 11). Therefore, we believe that our method would be a reliable and effective choice for WMH segmentation in clinical practice.

Our system provided a visual interface for WMH segmentation that integrated the functions of displaying the segmentation map and the value of each subclassified WMH volume. Previous studies have demonstrated that WMH in different locations may have distinctive etiologies, histopathologies and substantial functional relevance. Both are considered to be probable ischemic-origin WMH, PWMH may be more associated with cognitive impairment than DWMH (De Groot et al., 2002; Godin et al., 2010; Zhu et al., 2019). In addition to ischemic WMH, JVWMH and JCWMH may have non-ischemic origins and functional correlates (Kim et al., 2008). In particular, JVWMH, which is very close to the ventricle and is commonly seen in most elderly people, likely derives from CSF leakage and has different clinical relevance from PWMH (Zijdenbos et al., 2002; Kim et al., 2008; Li et al., 2019). There is still a lack of uniform standards for the division of WMH distribution, and the segmentation results of subclassified WMH conducted by our algorithm were achieved in strict accordance with the distance from the lateral ventricle, which would inevitably have some limitations. Nevertheless, the segmentation map and quantitative assessment of these subclassified WMH provided by our system would still be promising to further determine the etiology and clinical value of WMH progression and distribution.

Several complementary measures were conducted to further validate the generalization and robustness of our methods. First, manual segmentation is imperfect; in this sense, the use of several manual segmentations can improve the validation by creating an improved silver standard (García-Lorenzo et al., 2013), for which we constructed a silver standard dataset including patients with mild to severe WMH lesion loads. The test results on the silver standard dataset showed that our method and constructed dataset were effective and robust. Furthermore, in addition to the datasets with thick-slice MR images, further analysis on the 2017 WMH Segmentation Challenge (Kuijf et al., 2019) dataset (with thin slices of 3 mm) also showed an excellent performance (very close to the 1st ranked algorithm using 3D CNN and better than the remaining methods in the Challenge), indicating that the 2D VB-Net we proposed could be applied to MR images with various imaging protocols (Basile et al., 2006). Finally, in terms of the WMH volume-Fazekas correlations, our method was as good as manual delineation, and the consistency of the proposed algorithm with manual delineations was overall better than those from other competing methods (especially for DWMH), further indicating that the proposed automatic method can be a valid substitute for manual segmentation of WMH for clinical practice and research.

Some limitations should also be addressed. Primarily, the accuracy of segmentation was significantly better for WMH than for other coexisting intracranial lesions, although the relatively high values of lesion F1 for these other lesions indicated effective segmentation. This result was mainly due to the lower quality of the labeled ground truth of these pathologies compared to that of the WMH lesions, since these lesions were sometimes more difficult to identify (especially PVS in thick-slice MR images) or the boundaries were too obscure to delineate; semi-automated tools concentrated more on WMH segmentation and could not be used to help improve the consistency of delineation. Second, given the difficulty of the WMH segmentation task, there is still room for the algorithm to reach the ground truth generated by two experienced observers with the proposed labeling strategies. Nevertheless, compared to the previous state-of-the-art fully automatic methods and independent manual delineation (even though assisted by semi-automatic tools), our proposed algorithm demonstrated very competitive performance and general application. Notably, the recall of the “definite” WMH by our proposed 2D VB-Net reached a value over 0.87 on the independent datasets (0.873 on IDS 1 and 0.878 on IDS 2), which indicated an even better performance on the lesion regions commonly delineated by both observers. Third, it should be noted that current recommendations for brain imaging in some disorders, such as MS (Sastre-Garriga et al., 2020) and brain tumors (Kaufmann et al., 2020), clearly stated that images do not have to be acquired with thickness greater than 3 mm. However, our algorithm also achieved excellent performance for WMH segmentation on thin-slice MR images. Fourth, in consideration of the very thick layer and the strong partial volume effect, it is difficult to say that there is only a single component of tissue in a certain voxel. For these reasons and the possibly missed CSF suppression on FLAIR images, WMH would be judged only when the signals of all the sequences fulfilled the criteria of WMH in the labeling process, which may cause the volumes of WMH to be conservatively estimated. Finally, dynamic observations in large-scale studies are still warranted to determine the detailed role of WMH in the progression of various disorders using our method and further verify the clinical value of the algorithm in the future.

In conclusion, we proposed a novel 2D VB-Net algorithm for automated WMH segmentation based on a very large dataset with 2D thick-slice MR images that correspond to the protocol more frequently applied in clinical practice. The method not only showed a high segmentation accuracy but also provided information on the subclassification of WMH and could identify and segment other coexisting intracranial lesions well, which was validated on both the 2017 MICCAI WMH Segmentation Challenge (with thin MR slices) and clinical data. The study demonstrates that the proposed 2D VB-Net is a multi-modal, robust algorithm for WMH segmentation in multiple application scenarios and can be a promising tool in clinical practice.

## Data availability statement

The datasets presented in this study can be found in online repositories. The names of the repository/repositories and accession number(s) can be found below: https://github.com/haohaohuang-tj/WMH and https://github.com/simonsf/wmh-segmentation.

## Ethics statement

The studies involving human participants were reviewed and approved by The Ethics Committee of Tongji Hospital, Tongji Medical College, Huazhong University of Science and Technology. The patients/participants provided their written informed consent to participate in this study. Written informed consent was obtained from the individual(s) for the publication of any potentially identifiable images or data included in this article.

## Author contributions

WZ, WW, SX, and XL conceived and designed the study. WZ, HH, WW, SX, and XL contributed to the acquisition of the data. WZ, HH, YZ, FS, HS, RC, RH, and SX contributed to the analysis and interpretation of the data. YZ, FS, HS, RC, and RH provided technical support. WZ, HH, YZ, and XL drafted the manuscript. FS, HS, SX, and XL reviewed the manuscript. XL supervised the projects. All authors read and approved the final version of the manuscript.

## Footnotes

1
www.R-project.org
