# Supplementary material for: Automatic segmentation of white matter hyperintensities in routine clinical brain MRI by 2D VB-Net: A large-scale study
Source: Front Aging Neurosci. 2022 Jul 29;14:915009. doi: 10.3389/fnagi.2022.915009 (PMC9372352; doi:10.3389/fnagi.2022.915009)
Supplement: Supplementary file 1 [file Data_Sheet_1.pdf]

Supplementary Material for

Automatic segmentation of white matter hyperintensities in routine clinical brain MRI by 2D VB-Net: A large-scale study

Supplementary Table 1. MRI acquisition protocols.

	Parameter (unit)	UIH uMR 780	GE Signa HDxt	GE Discovery MR750	GE Signa Excite	GE Signa HDxt	GE Signa Creator	GE Brivo MR355
	Magnetic field strength (T)	3.0	3.0	3.0	1.5	1.5	1.5	1.5
FLAIR	TR/TE/TI (ms)	8000/106/2425	8002/170/2100	8400/115/2200	8602/120/2100	8802/122/2200	8400/102/2100	8000/131/2100
	Pixel spacing (mm)	0.5047 * 0. 5047	0.4688 * 0.4688	0.4688 * 0.4688	0.4688 * 0.4688	0.4688 * 0.4688	0.4688 * 0.4688	0.4688 * 0.4688
	Inter-slice gap (mm)	1.5	2	2	2	2	2	2
	Slice thickness (mm)	5.5	6	6	6	6	6	6
	Matrix	456 * 396	512 * 512	512 * 512	512 * 512	512 * 512	512 * 512	512 * 512
	No. of slices	18	16	16	16	16	16	16
T1-weighted	TR/TE/TI (ms)	1800/11/790	1785/24/720	1750/24/780	2120/12/700	2180/11/720	2430/19/750	2311/20/750
	Pixel spacing (mm)	0.5047 * 0. 5047	0.4688 * 0.4688	0.4688 * 0.4688	0.4688 * 0.4688	0.4688 * 0.4688	0.4688 * 0.4688	0.4688 * 0.4688
	Inter-slice gap (mm)	1.5	2	2	2	2	2	2
	Slice thickness (mm)	5.5	6	6	6	6	6	6
	Matrix	456 * 396	512 * 512	512 * 512	512 * 512	512 * 512	512 * 512	512 * 512
	No. of slices	18	16	16	16	16	16	16
T2-weighted	TR/TE (ms)	4000/93	3800/120	3800/105	4220/103	3280/103	3750/113	3820/116
	Pixel spacing (mm)	0.5047 * 0. 5047	0.4688 * 0.4688	0.4688 * 0.4688	0.4688 * 0.4688	0.4688 * 0.4688	0.4688 * 0.4688	0.4688 * 0.4688
	Inter-slice gap (mm)	1.5	2	2	2	2	2	2
	Slice thickness (mm)	5.5	6	6	6	6	6	6
	Matrix	456 * 396	512 * 512	512 * 512	512 * 512	512 * 512	512 * 512	512 * 512
	No. of slices	18	16	16	16	16	16	16

Supplementary Table 2. Subject distribution across imaging devices.

Scanner	Number	Total
GE Signa HDxt 3.0 T	471	1045
GE Discovery MR750 3.0 T		
UIH uMR 780 3.0 T		
GE Signa Excite 1.5 T	574	
GE Signa HDxt 1.5 T		
GE Signa Creator 1.5 T		
GE Brivo MR355 1.5 T		

Supplementary Table 3. Detailed parameters of the proposed 2D VB-Net.

Blocks	Subblocks or layers	Input dimensions	Output dimensions
Input block	Conv(k=3,p=1,s=1)+BN+ReLU	256*256*3	256*256*16
Down block1(32)	Conv(k=2,p=0,s=2)+BN+ReLU	256*256*16	128*128*32
	Conv(k=3,p=1,s=1)+BN+ReLU*	128*128*32	128*128*32
	Conv(k=3,p=1,s=1)+BN*	128*128*32	128*128*32
	ReLU*	128*128*32	128*128*32
Down block2(64)	Conv(k=2,p=0,s=2)+BN+ReLU	128*128*32	64*64*64
	Conv(k=1,p=0,s=1)+BN+ReLU*	64*64*64	64*64*16
	Conv(k=3,p=1,s=1)+BN+ReLU*	64*64*16	64*64*16
	Conv(k=1,p=0,s=1)+BN+ReLU*	64*64*16	64*64*64
	Conv(k=1,p=0,s=1)+BN+ReLU*	64*64*64	64*64*16
	Conv(k=3,p=1,s=1)+BN+ReLU*	64*64*16	64*64*16
	Conv(k=1,p=0,s=1)+BN*	64*64*16	64*64*64
	ReLU*	64*64*64	64*64*64
Down block3(128)	Conv(k=2,p=0,s=2)+BN+ReLU	64*64*64	32*32*128
	Conv(k=1,p=0,s=1)+BN+ReLU*	32*32*128	32*32*32
	Conv(k=3,p=1,s=1)+BN+ReLU*	32*32*32	32*32*32
	Conv(k=1,p=0,s=1)+BN+ReLU*	32*32*32	32*32*128
	Conv(k=1,p=0,s=1)+BN+ReLU*	32*32*128	32*32*32
	Conv(k=3,p=1,s=1)+BN+ReLU*	32*32*32	32*32*32
	Conv(k=1,p=0,s=1)+BN*	32*32*32	32*32*128
	ReLU*	32*32*128	32*32*128
Down block3(256)	Conv(k=2,p=0,s=2)+BN+ReLU	32*32*128	16*16*256
	Conv(k=1,p=0,s=1)+BN+ReLU*	16*16*256	16*16*64
	Conv(k=3,p=1,s=1)+BN+ReLU*	16*16*64	16*16*64
	Conv(k=1,p=0,s=1)+BN+ReLU*	16*16*64	16*16*256
	Conv(k=1,p=0,s=1)+BN+ReLU*	16*16*256	16*16*64
	Conv(k=3,p=1,s=1)+BN+ReLU*	16*16*64	16*16*64
	Conv(k=1,p=0,s=1)+BN*	16*16*64	16*16*256
	ReLU*	16*16*256	16*16*256
Up block1(256)	Conv(k=2,p=0,s=2)+BN+ReLU	16*16*256	32*32*128
	SkipConv(k=3,p=1,s=1)+BN+ReLU*	32*32*128	32*32*128
	Conv(k=1,p=0,s=1)+BN+ReLU*	32*32*256	32*32*64
	Conv(k=3,p=1,s=1)+BN+ReLU*	32*32*64	32*32*64
	Conv(k=1,p=0,s=1)+BN+ReLU*	32*32*64	32*32*256
	Conv(k=1,p=0,s=1)+BN+ReLU*	32*32*256	32*32*64
	Conv(k=3,p=1,s=1)+BN+ReLU*	32*32*64	32*32*64
	Conv(k=1,p=0,s=1)+BN*	32*32*64	32*32*256
	ReLU*	32*32*256	32*32*256
Up block2(128)	Conv(k=2,p=0,s=2)+BN+ReLU	32*32*256	64*64*64

	SkipConv(k=3,p=1,s=1)+BN+ReLU*	64*64*64	64*64*64
	Conv(k=1,p=0,s=1)+BN+ReLU*	64*64*128	64*64*32
	Conv(k=3,p=1,s=1)+BN+ReLU*	64*64*32	64*64*32
	Conv(k=1,p=0,s=1)+BN+ReLU*	64*64*32	64*64*128
	Conv(k=1,p=0,s=1)+BN+ReLU*	64*64*128	64*64*32
	Conv(k=3,p=1,s=1)+BN+ReLU*	64*64*32	64*64*32
	Conv(k=1,p=0,s=1)+BN*	64*64*32	64*64*128
	ReLU*	64*64*128	64*64*128
Up block3(64)	Conv(k=2,p=0,s=2)+BN+ReLU	64*64*128	128*128*32
	SkipConv(k=3,p=1,s=1)+BN+ReLU*	128*128*32	128*128*32
	SkipConv(k=3,p=1,s=1)+BN+ReLU*	128*128*32	128*128*32
	Conv(k=3,p=1,s=1)+BN+ReLU*	128*128*64	128*128*64
	Conv(k=3,p=1,s=1)+BN*	128*128*64	128*128*64
	ReLU*	128*128*64	128*128*64
Up block4(32)	Conv(k=2,p=0,s=2)+BN+ReLU	256*256*64	256*256*16
	SkipConv(k=3,p=1,s=1)+BN+ReLU*	256*256*16	256*256*16
	SkipConv(k=3,p=1,s=1)+BN+ReLU*	256*256*16	256*256*16
	Conv(k=3,p=1,s=1)+BN+ReLU*	256*256*32	256*256*32
	Conv(k=3,p=1,s=1)+BN*	256*256*32	256*256*32
	ReLU*	256*256*32	256*256*32
Out_block	Conv(k=3,p=1,s=1)+BN+ReLU	256*256*32	256*256*16
	Conv(k=3,p=1,s=1)+BN+ReLU	256*256*16	256*256*16
	Conv(k=3,p=0,s=1)	256*256*16	256*256*3
	Softmax	256*256*3	256*256*1

k, kernel size; p, padding; s, stride. "*" denotes that these layers are residual units.

Supplementary Table 4. Performance of the algorithms on the multi-scanner dataset.

Lesion	Automatic method	Dice	*Recall*	Recall of definite WMH	*Precision*	*Hausdorff* ↓	*AVD* ↓	Lesion recall	Lesion F1
WMH	2D VB-Net_multi	0.792	0.815	0.883	0.798	8.927	23.158	0.783	0.702
	2D VB-Net_S	0.789**	0.830	0.894	0.776	9.460	24.287	0.791	0.694**
	uResNet	0.777**	0.864	0.718	0.724	15.484**	28.912**	0.856	0.633**
	3D V-Net	0.703**	0.771	0.827	0.661	7.809**	26.264*	0.766	0.671**
	VGGNet	0.766**	0.823	0.738	0.737	26.926**	25.132	0.822	0.625**
Other pathologies	2D VB-Net_multi	0.516	0.575	-	0.536	34.170	130.255	0.748	0.622
	2D VB-Net_S	0.220**	0.284	-	0.232	46.602**	282.220**	0.345	0.230**
	uResNet	0.424**	0.566	-	0.401	45.316**	276.756**	0.797	0.536**
	3D V-Net	0.474**	0.568	-	0.449	41.876**	183.39	0.764	0.611
	VGGNet	0.479**	0.593	-	0.461	38.929**	170.43	0.78	0.571*

*, ** *P* value of two-tailed paired *t*-test between the performance of 2D VB-Net _multi and the other algorithms;

*, $P < 0.05$ significance level, **, $P < 0.001$ significance level.

Supplementary Table 5. Performance of the algorithms on the two independent datasets.

Lesion	Automatic method	Dice	*Recall*	Recall of definite WMH	*Precision*	*Hausdorff* ↓	*AVD* ↓	Lesion recall	Lesion F1
Performance on IDS 1									
WMH	2D VB-Net_multi	0.775	0.808	0.873	0.764	8.057	21.297	0.822	0.724
	uResNet	0.726**	0.861	0.667	0.651	18.911**	46.382**	0.863	0.602**
	3D V-Net	0.647**	0.714	0.773	0.624	9.422*	36.827**	0.724	0.633**
	VGGNet	0.712**	0.835	0.660	0.646	29.166**	45.254**	0.851	0.578**
Other pathologies	2D VB-Net_multi	0.450	0.477	-	0.510	37.445	111.253	0.680	0.622
	uResNet	0.344**	0.428	-	0.387	46.195**	740.905*	0.680	0.526**
	3D V-Net	0.294**	0.358	-	0.307	51.637**	304.313*	0.542	0.434**
	VGGNet	0.400*	0.466	-	0.424	38.768	299.475*	0.689	0.571**
Performance on IDS 2									
WMH	2D VB-Net_multi	0.789	0.809	0.878	0.792	9.777	22.163	0.794	0.706
	uResNet	0.751**	0.833	0.704	0.713	20.965**	34.770**	0.834	0.587**
	3D V-Net	0.697**	0.75	0.812	0.673	8.677	28.267**	0.759	0.671**
	VGGNet	0.737**	0.824	0.696	0.695	33.05**	37.643**	0.825	0.573**
Other pathologies	2D VB-Net	0.495	0.540	-	0.539	35.931	94.207	0.718	0.626
	uResNet	0.367**	0.505	-	0.398	46.579**	289.562*	0.750	0.524**
	3D V-Net	0.362**	0.412	-	0.399	45.713*	109.252	0.622	0.512**
	VGGNet	0.469	0.544	-	0.485	38.055	108.321	0.749	0.573**

IDS, independent dataset.

*, ** *P* value of two-tailed paired *t*-test between the performance of 2D VB-Net _multi and the other algorithms;

*, $P < 0.05$ significance level, **： $P < 0.001$ significance level.

Supplementary Table 6. Average Dice performance of the algorithms for different WMH lesion load levels on the two independent datasets.

Dataset	Automatic method	Dice		
		< 5 ml	5–15 ml	> 15 ml
IDS 1	2D VB-Net_multi	0.695	0.785	0.847
	uResNet	0.616**	0.730**	0.837*
	3D V-Net	0.551**	0.638**	0.762**
	VGGNet	0.591**	0.729**	0.829**
IDS 2	2D VB-Net_multi	0.732	0.792	0.854
	uResNet	0.655**	0.768**	0.854
	3D V-Net	0.626**	0.704**	0.777**
	VGGNet	0.630**	0.752**	0.854

IDS, independent dataset.

*, ** P value of two-tailed paired t-test between 2D VB-Net _multi and the other algorithms;

*, $P < 0.05$ significance level, **, $P < 0.001$ significance level.

Supplementary Table 7. Characteristics of the 2017 MICCAI WMH Challenge publicly available dataset.

Datasets	Scanner name	Voxel spacing (mm)	Number
Utrecht	3T Philips Achieva	0.96 * 0.95 * 3.00	20
Singapore	3T Siemens TrioTim	1.00 * 1.00 * 3.00	20
GE3T	3T GE Signa HDxt	0.98 * 0.98 * 1.20	20

Supplementary Table 8. Correlation analysis of WMH volumes extracted from manual delineation and algorithms based on a widely used definition with visual rating scores and comparisons between correlation coefficients.

	Total WMH			PWMH			DWMH		
	r	Δ*r*	95% Bootstrap CI of Δ*r*	*r*	Δ*r*	95% Bootstrap CI of Δ*r*	*r*	Δ*r*	95% Bootstrap CI of Δ*r*
Manual delineation	0.895			0.854			0.840		
2D VB-Net	0.900	-0.005	(-0.0251, 0.0132)	0.868	-0.014	(-0.0285, 0.0053)	0.831	0.009	(-0.0277, 0.0401)
uResNet	0.890	0.005	(-0.0221, 0.0309)	0.868	-0.014	(-0.0367, 0.0149)	0.813	0.027	(0.0110, 0.0705)
3D V-Net	0.872	0.023	(-0.0043, 0.0571)	0.855	-0.001	(-0.0237, 0.0295)	0.781	**0.059**	**(0.0086, 0.1167)**
VGGNet	0.868	0.027	(-0.0003, 0.0678)	0.864	-0.010	(-0.0317, 0.0178)	0.767	**0.073**	**(0.0267, 0.1353)**

According to the criteria proposed by Fazekas et al., the total WMH were subdivided into only 2 part: the PWMH and the DWMH. Here we adopted a widely used rule (PWMH were defined as the WMH within 10 mm from the lateral ventricle, otherwise as DWMH) to further segment WMH.

r refers to Spearman’s correlation coefficient of WMH volumes extracted from manual delineations or algorithms with corresponding Fazekas visual rating scores. Δ*r* refers to the difference between Spearman’s correlation coefficient of manual-annotated WMH volumes with Fazekas scores and Spearman’s correlation coefficient of WMH volumes extracted from each automatic algorithm with Fazekas scores, using a Bootstrap method for 1,000 bootstrapping times. Bold values represent a significance of Δ*r* that is defined by a 95% Bootstrap CI entirely above or below 0 (uncorrected, considering that the comparative analysis is exploratory).

CI, confidence interval; DWMH, deep white matter hyperintensities; PWMH, periventricular white matter hyperintensities.

We hope this explanation and the revised version of the manuscript are clearer.

Supplementary Table 9. Performance of 2D VB-Net on the silver standard dataset.

Lesions	Dice	*Recall*	*Precision*	*Hausdorff* ↓	*AVD* ↓	Lesion recall	Lesion F1
WMH	0.782	0.887	0.728	10.266	44.635	0.853	0.642
Other pathologies	0.543	0.734	0.468	40.621	88.854	0.908	0.631

Supplementary Table 10. White matter lesion load of each data point on the silver standard dataset and the Dice value of the algorithm.

Subject	White matter lesion load (ml)	Dice
1	36.41	0.875
2	2.68	0.713
3	2.10	0.564
4	3.38	0.727
5	6.24	0.845
6	38.44	0.849
7	28.15	0.889
8	15.91	0.824
9	121.12	0.933
10	7.46	0.824
11	10.03	0.795
12	34.49	0.851
13	6.28	0.769
14	11.45	0.839
15	53.48	0.913
16	25.48	0.854
17	7.99	0.695
18	2.39	0.741
19	0.82	0.298
20	21.62	0.837

The algorithm showed better performance on patients with larger lesions, but even for patients with small lesions, the average Dice value reached 0.7.

Supplementary Table 11. Performance of the proposed fully automatic method and semi-automatic method for WMH on the silver standard dataset.

Method	Dice	*Recall*	*Precision*	*Hausdorff* ↓	*AVD* ↓	Lesion recall	Lesion F1
2D VB-Net	0.782	0.887	0.728	10.266	44.635	0.853	0.642
Semi-automatic method*	0.762	0.695	0.893	11.245	32.382	0.635	0.717

*The performance of the semi-automatic method refers to manual delineations using semi-automatic tools by an experienced observer independently.

Supplementary Table 12. Summary of recent works on automated WMH segmentation.

Automatic method	Dataset Size	Modality (spacing, mm) [1]	Dice [2]	
			WMH	Other pathologies
Jain et al., 2015	20	FLAIR (0.98*0.98*1.2)/T1 (0.94*0.94*1)	0.67	-
Guerrero et al., 2018	167	FLAIR (0.94*0.94*6.5)	0.69	0.40
Li et al., 2018	110	FLAIR (1.0*1.0*3.0)/T1 (1.0*1.0*3.0)	0.80	-
Valverde et al., 2017	35	FLAIR (0.49*0.49*3)/T1 (1.0*1.0*1.2)	0.535	-
Present method	849	FLAIR (0.5*0.5*8)/T1 (0.5*0.5*8)/T2 (0.5*0.5*8)	0.792	0.516

[1] Part of the work has multiple data acquisition protocols; typical values are listed here.

[2] Only studies that reported Dice values of algorithms are listed here. Part of the work was performed using multiple datasets for performance testing; typical values are listed here.

Supplementary Table 13. The internal storage size and the time consumption for one case of the algorithms in the task of WMH segmentation.

Algorithm	2D VB-Net	uResNet	3D V-NET	VGGNet
Internal storage size (M)	1.08	30.85	11.35	82.36
Time consumption (s)	1.38	1.03	13.96	2.56

Supplementary Table 14. Impact of resampling spacing and crop size on network performance.

Automatic method	Spacing (mm)	Patch Size	Dice	
			WMH	Other pathology
2D VB-Net	0.5*0.5	256*256	0.795	0.521
	0.5*0.5	128*128	0.761	0.334
	1*1	256*256	0.745	0.427
uResNet	0.5*0.5	256*256	0.785	0.484
	0.5*0.5	128*128	0.753	0.336
	1*1	64*64	0.721	0.272
3D V-Net	0.5*0.5*5	256*256*16	0.703	0.474
	0.5*0.5*5	128*128*16	0.652	0.391
VGGNet	0.5*0.5	256*256	0.788	0.486
	0.5*0.5	128*128	0.758	0.403

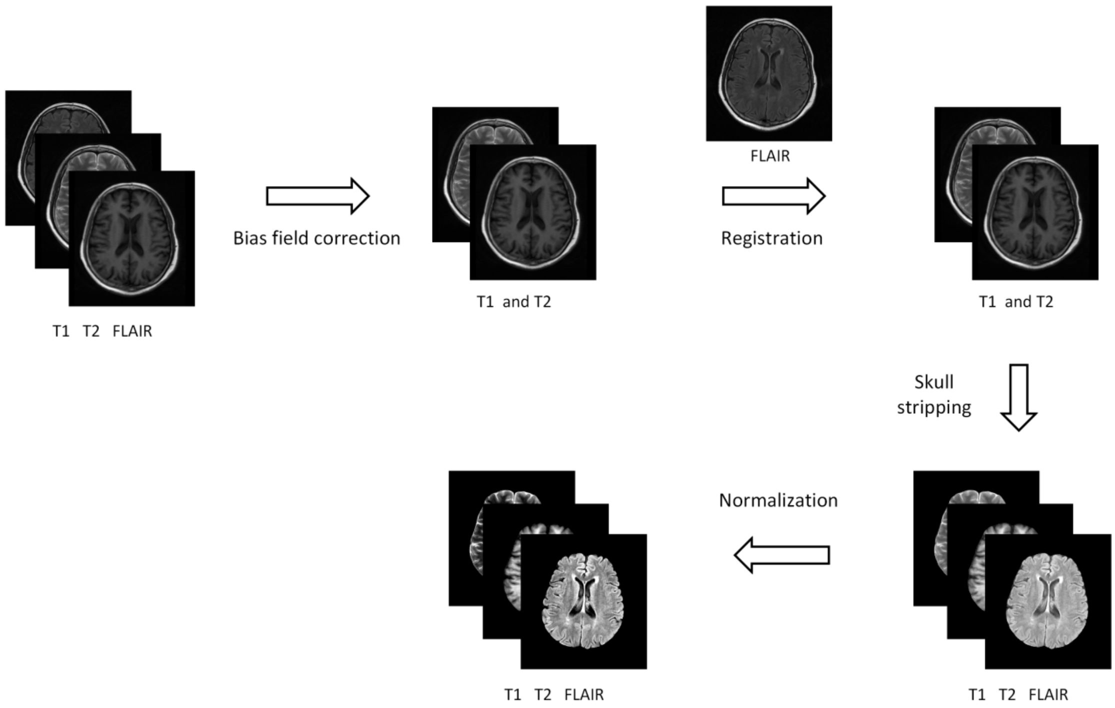

Supplementary Figure 1. Image preprocessing pipeline.

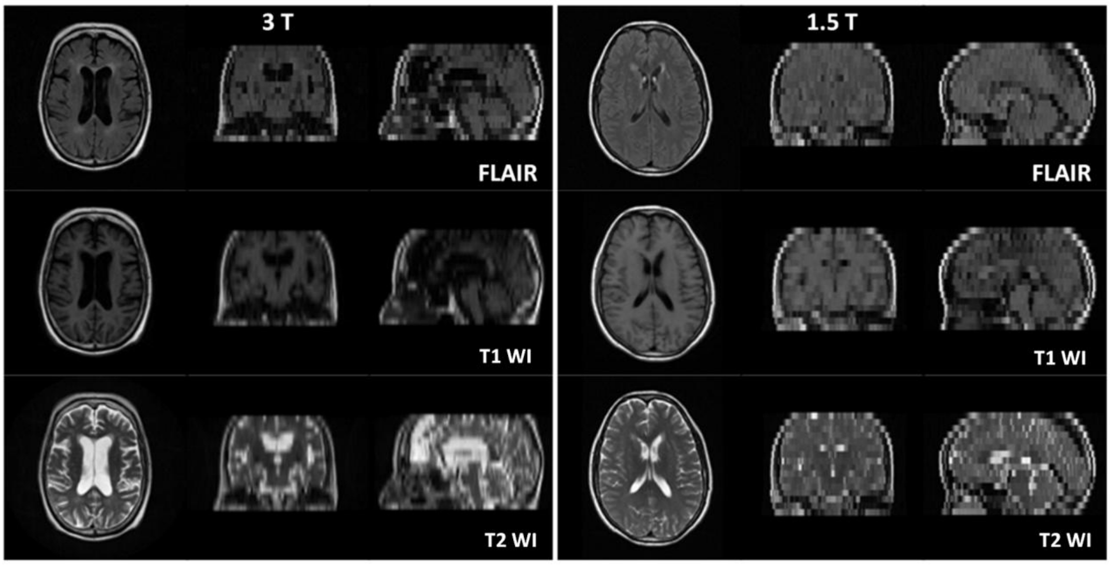

Supplementary Figure 2. The data used in this work from different views and modalities.

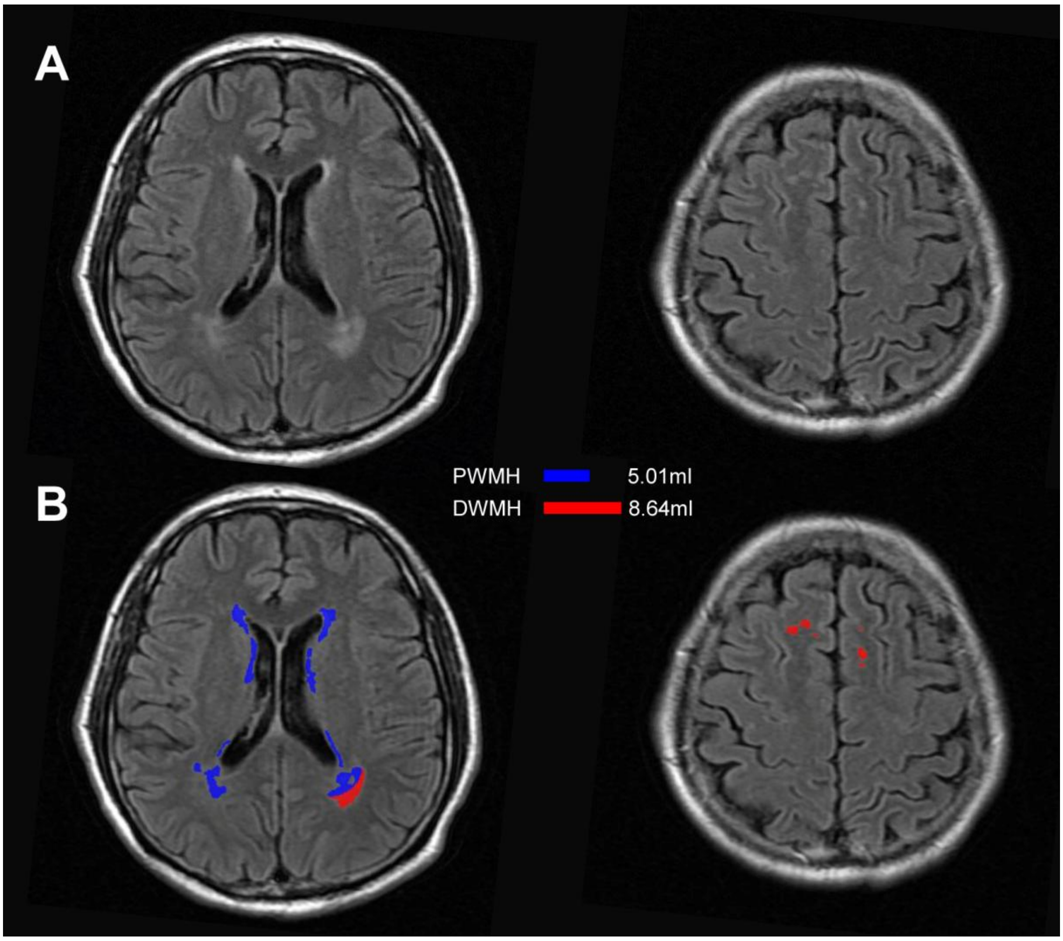

Supplementary Figure 3. An example of segmentation results for subclassified WMH according to a widely used rule. (A) FLAIR images; (B) display of the segmentation map and the volumes of each subclassified WMH area according to a widely used rule (PWMH were defined as the WMH within 10 mm from the lateral ventricle, otherwise as DWMH). The regions of each subclassified WMH are represented by different colors. DWMH, deep white matter hyperintensities; PWMH, periventricular white matter hyperintensities.

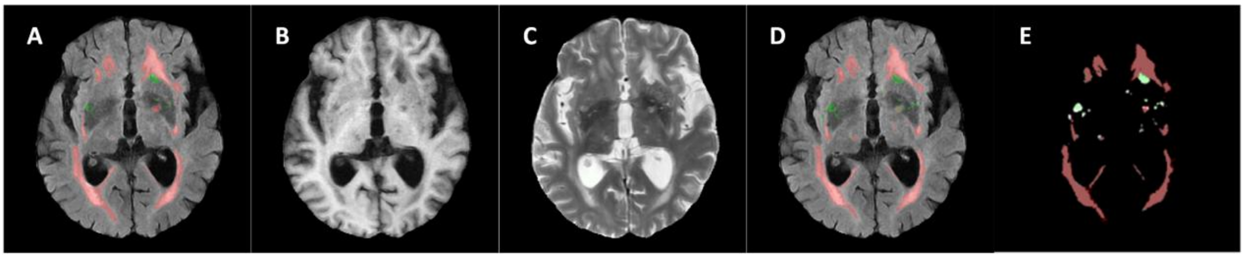

Supplementary Figure 4. The best case in the silver standard dataset. (A) FLAIR image with ground truth overlaid, (B) T1-weighted image, (C) T2-weighted image, (D) FLAIR image overlaid with the segmentation results by the 2D VB-Net algorithm, (E) the ground truth with the resulting masks produced by the 2D VB-Net algorithm. The figure shows the best case in the silver standard dataset for WMH segmentation, in which the 2D VB-Net achieved a Dice value of 0.933, a *recall* value of 0.917, and a *precision* value of 0.951.

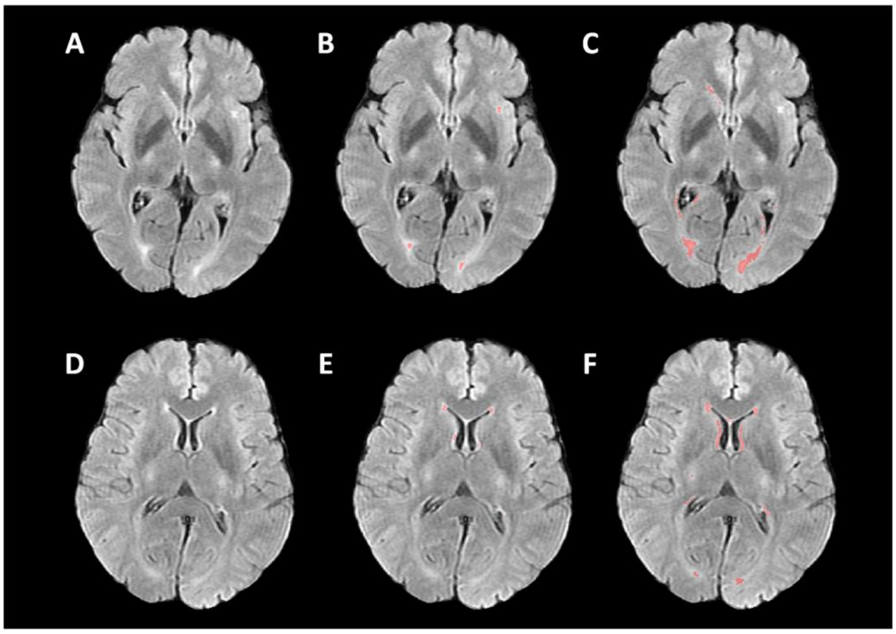

Supplementary Figure 5. The worst case on the silver standard dataset. (A) (D) FLAIR image, (B) (E) FLAIR image with ground truth overlaid, (C) (F) FLAIR image overlaid with the segmentation results by the 2D VB-Net algorithm. The first row shows the 9th slice of the FLAIR images, and the second row shows the similarity but for the 10th slice. The figure shows the worst case in the silver standard dataset for WMH segmentation, in which the 2D VB-Net algorithm achieved a Dice value of 0.298, a recall value of 0.940, and a precision value of 0.177.

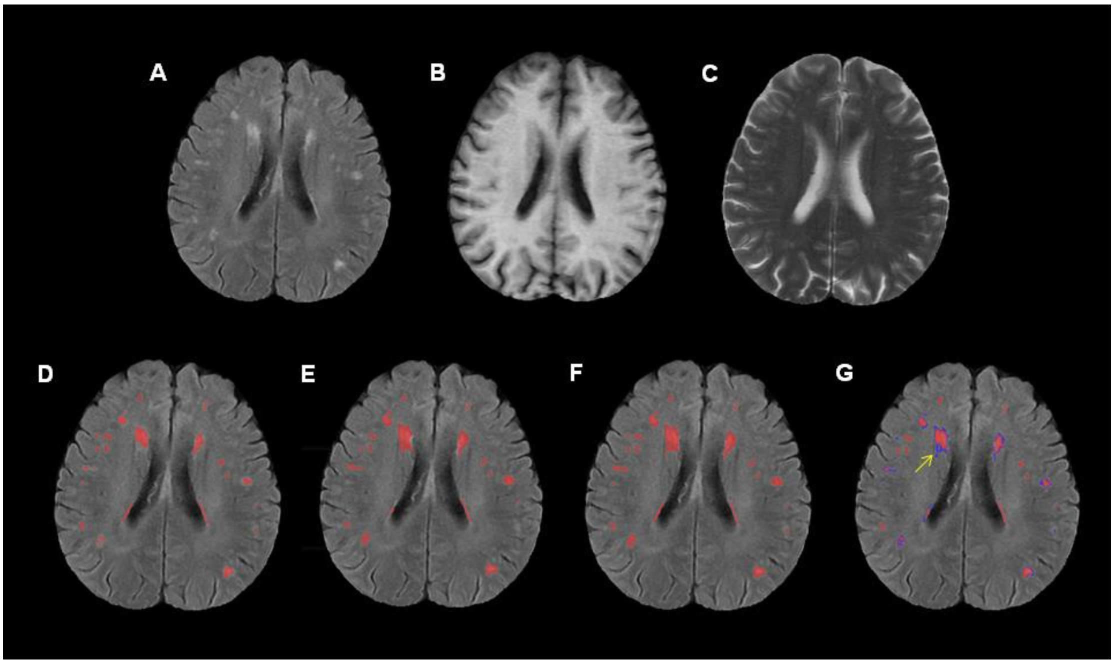

Supplementary Figure 6. Handling inter-observer variations. (A) FLAIR image, (B) T1-weighted image, (C) T2-weighted image, (D) (E) manual delineations by two observers, (F) the union of the two delineations as the ground truth, (G) red areas indicate the intersections of the two delineations, defined as “definite” WMH regions. Blue regions indicate discrepant areas, defined as “suspected” WMH regions. The yellow arrow points to the small WMH region as “suspected” WMH.
